# Topology‐Optimized Bound States in the Continuum with High‐Q Acoustic Field Enhancement

**DOI:** 10.1002/advs.202414344

**Published:** 2025-03-27

**Authors:** Weibai Li, Kazem Ghabraie, Xiaodong Huang

**Affiliations:** ^1^ School of Engineering Swinburne University of Technology Hawthorn VIC 3122 Australia; ^2^ School of Engineering Deakin University Waurn Ponds VIC 3216 Australia

**Keywords:** acoustic metamaterials, bound states in the continuum, high‐Q resonances, topology optimization

## Abstract

Achieving high‐quality (high‐Q) acoustic resonances remains a critical goal in acoustic device design, given their exceptional sound manipulation capabilities. However, enhancing Q‐factors is often hindered by energy dissipation and material losses, except for leveraging bound states in the continuum (BICs). This paper introduces a methodology utilizing topology optimization to achieve high‐Q resonances based on the concept of BICs, which effectively confine acoustic waves by minimizing energy leakage. This method explores entirely new topology classes through the optimization of a single unit cell embedded within periodic arrays. By engineering quasi‐BIC modes and experimentally validating sharp pressure field enhancements, a robust technique that enables precise tuning of resonance frequencies and improves resilience against external perturbations, which is challenging for the conventional parameter‐tuning approach is presented. These findings show promise for advancing wave‐confining applications, such as energy harvesting and acoustic filtering, while pushing the performance boundaries of acoustic devices.

## Graphical abstract

This study presents a topology optimization method for acoustic metamaterials, achieving high‐Q resonances by redesigning a single unit cell within a periodic array. The design enables precise pressure confinement and energy trapping, validated through experiments. The approach highlights potential applications in advanced acoustic devices, including filters, isolators, and energy harvesters.

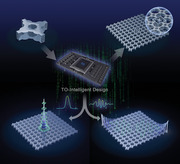

## 1 Introduction

Achieving high‐quality (high‐Q) acoustic resonances is essential for numerous advanced applications, including enhanced sensitivity in acoustic sensors, improved performance in ultrasonic imaging, and superior sound manipulation in acoustic metamaterials.^[^ ^1^, ^2^, ^3^ ^]^ The quality factor (Q‐factor) is a critical measure of the overall loss in a resonant system, where higher values indicate lower loss and superior wave‐trapping abilities. A promising method for high‐Q wave confinement is through bound states in the continuum (BICs). BICs are non‐radiating modes embedded within continuous spectra, characterized by exceptionally low structural loss, even in open systems.^[^ ^4^ ^]^ First introduced in quantum mechanics,^[^ ^5^ ^]^ BICs have since been extended to various wave systems, including acoustic,^[^ ^6^, ^7^, ^8^, ^9^, ^10^, ^11^, ^12^ ^]^ elastic,^[^ ^13^, ^14^ ^]^ photonic,^[^ ^15^, ^16^, ^17^ ^]^ dielectric,^[^ ^18^, ^19^, ^20^ ^]^ and plasmonic systems.^[^ ^21^, ^22^, ^23^ ^]^ Unlike traditional bandgap‐confined resonances, which are limited by finite Q‐factors, BICs theoretically exhibit infinite Q‐factors due to zero radiative losses. In practical implementations, BICs manifest as quasi‐BICs, which retain extremely high Q‐factors despite being finite, making them ideal for applications such as absorptions,^[^ ^24^ ^]^ lasers,^[^ ^25^, ^26^, ^27^ ^]^ sensors,^[^ ^28^, ^29^, ^30^ ^]^ waveguides,^[^ ^31^ ^]^ and nonlinearity enhancement.^[^ ^32^, ^33^, ^34^ ^]^

Distinctive from bandgap‐confined states, solely relying on the absence of radiative pathways within a spectral gap, BICs achieve energy confinement within the radiation continuum through physical mechanisms such as symmetry mismatch, parameter tuning, or destructive interference between radiative modes.^[^ ^4^, ^35^ ^]^ The characteristics of BICs could offer capabilities beyond the limitations of bandgap‐confined states. Recently, BICs have been extensively studied and experimentally demonstrated in open systems, revealing their versatility and potential for diverse applications.^[^ ^36^, ^37^, ^38^, ^39^, ^40^, ^41^ ^]^ Most studies on BICs contribute to confinement in one dimension to reduce the spatial extension of the field, such as confinement to a 2D slab in a 3D system, a 1D line in a 2D system, or a 0D cavity in a 1D system. A summary of existing studies on dimensional transitions for BIC confinement is provided in **Table** **1**.

**Table 1:** Studies on BIC confinement across different dimensional transitions.

Dimensional confinement	Description and represented references
3D → 2D	Confinement of waves to a 2D slab within a 3D system.^[^ ^8^, ^18^, ^19^, ^20^, ^21^, ^22^, ^23^, ^40^, ^41^ ^]^
2D → 1D	Confinement to a line or waveguide within a 2D system.^[^ ^13^, ^16^, ^17^ ^]^
1D → 0D	Localization to a cavity within a 1D waveguide.^[^ ^6^, ^9^, ^12^, ^14^, ^24^, ^35^, ^37^, ^39^ ^]^
3D → 0D	Full localization to a point‐like cavity in a 3D system.^[^ ^7^, ^42^, ^43^ ^]^
2D → 0D	Localization to a cavity within a 2D system.^[^ ^10^, ^11^, ^15^, ^25^, ^26^ ^]^

Despite significant progress has been made in the design and application of BICs, traditional approaches remain fundamentally limited by their reliance on physical analysis and parameter tuning case by case. It is essential to break through these limitations and develop a systematic, flexible, and versatile design tool, achieving the diversity and innovation of BIC structures supporting high‐Q resonances. This reminds us of whether we can use machine learning and generative algorithms to find BICs.^[^ ^44^, ^45^, ^46^, ^47^ ^]^ However, these artificial intelligence (AI)‐based approaches require large, high‐quality datasets for training, which are currently limited. Remaining tethered to the boundaries of the training data, AI also struggled to create new and unique BIC structures from scratch.^[^ ^48^ ^]^ Instead, state‐of‐the‐art topology optimization (TO) offers a robust and automated design methodology that does not rely on physical intuition,^[^ ^49^, ^50^ ^]^ and has demonstrated its capabilities in designing structural and multiphysics metamaterials with desired mechanical and functional properties.^[^ ^51^, ^52^, ^53^, ^54^, ^55^ ^]^ Although a gradient‐based TO method requires the trivial derivation of the optimization problem and sensitivity analysis, once successful, it offers the ability to explore a wide range of diverse topologies, free from limitations imposed by initial configurations.

This paper will meet the above need and develop a topology optimization approach to discover BIC structures without any need for human intervention. Our strategy is to minimize radiation leakage and enhance wave confinement by digitally redesigning a unit cell within the periodic lattices till it supports high‐Q resonances, underpinned by the concept of BICs. While the study focuses on confining a pressure field to a 0D cavity within a 2D system, the proposed approach is versatile and has the potential to extend to multi‐dimensional wave confinement in various physical systems. Additionally, the innovative topologies generated by TO can serve as a high‐quality dataset for AI models, paving the way for advanced data‐driven design tools.

## 2 Results and Discussion

**Figure 1:**
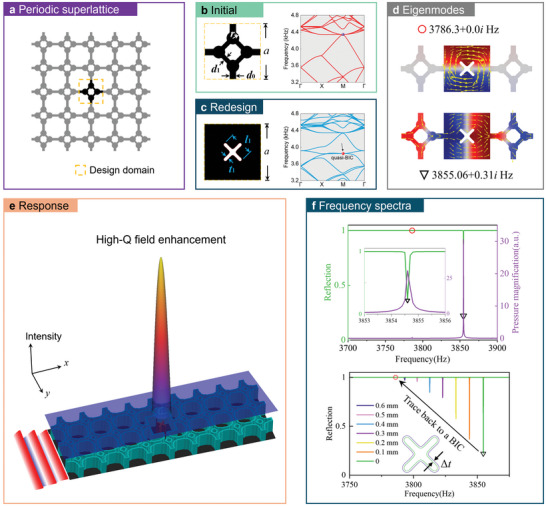
High‐Q resonances by TO. a) Periodic metamaterial constructed by a cavity‐tube unit cell. b) One unit cell in the initial supercell and the band diagram. The air domain is plotted in black color. c) The redesign central unit and the band diagram of the resulting supercell. d) Eigen‐field profiles of the high‐Q resonances at 3786.3 + 0.000158𝑖 Hz (red circle marker) and 3855.06 + 0.31*i* Hz (black triangle marker). The pressure field and the acoustic intensity field within the central unit are indicated by the color map and yellow arrow lines, respectively. e) Schematic of the acoustic energy confinement under far‐field excitation. f) Upper: reflection and pressure response to far‐field incident waves. Lower: reflection curves in the condition of the solid scatter size, which is tuned by the boundary offset value Δ*t* as illustrated by the inset.

Inspired by the BICs generated from defect modes at twofold degeneracies,^[^ ^10^, ^15^, ^25^ ^]^ our first example initiated the periodic metamaterial composing of a cavity‐tube lattice, as illustrated in **Figure** **1**a,b. This configuration features a lattice constant *a* = 40 mm and four identical circular cavities with a radius *r*_0_ = 4 mm. The width of the small channels are *d*_1_ = 3.36 mm and *d*_0_ = 4.6 mm, respectively. These parameters are selected to facilitate a twofold degeneracy at ≈4300 Hz. The band diagram for the initial supercell is shown in the right panel of Figure 1b. To account for energy dissipation due to the thermal and viscous losses and radiation to the outgoing boundaries, the eigenfrequencies for a finite structure can be computed as complex numbers, *ω_k_* = Re(*ω_k_*) + *i*Im(*ω_k_*). The positive imaginary part of the eigenfrequency corresponds to a time dependence given by a factor of *exp*(*iωt*) in numerical analysis.^[^ ^56^ ^]^ Thus, the Q‐factor of each eigenmode can be estimated by *Q*_ω_ = Re(*ω_k_*)/2Im(*ω_k_*).

To achieve 0D confinement with a high Q‐factor at the desired frequency (e.g., 3860 Hz), we propose a TO algorithm explained in Section 4.1., starting from the periodic lattice and redesigning the central unit cell shown in Figure 1a. The optimization aims at minimizing radiation leakage at the frequency ranging was set between 3827 to 3870 Hz. The filter radius is taken as the total length of three elements (≈0.94 mm), which makes the final design easily fabricated. The principle of choosing the filter radius refers to Section 4.1. The final design with the minimum radiation leakage is depicted in Figure 1c, featured with the dimensions of *t*_1_ = 2.38 mm and *l*_1_ = 17.12 mm. The band diagram of the supercell, incorporating the optimized unit cell as a point defect, displays continuous frequency bands and localized eigenmodes ≈3850 Hz. This indicates that the resulting resonant mode is a quasi‐BIC rather than being confined by a bandgap.

To verify the TO design's ability to support high‐Q resonances in a finite structure, we assemble the optimized structure into a 9 × 3 superstructure and perform simulations in COMSOL (See Figure 6 in Section 4.2.). It is noted that the initial structure does not support any localized modes within the frequency range of 3000 and 4200 Hz. Instead, the optimized superstructure produces two eigenmodes with significantly high Q‐factors. The local field profiles of these two modes are shown in Figure 1d. One resonant mode has a complex eigenfrequency of 3855.06 + 0.31*i* Hz, interacting with the far‐field radiation. Another mode, characterized by an extremely low imaginary component (3786.3 + 0.000158𝑖 Hz), shows highly localized pressure distribution and trapped intensity flow, suggesting the presence of a BIC that cannot be excited by far‐field incidence.

Figure 1e schematically illustrates the concentration of acoustic energy within the superstructure under a far‐field excitation. The reflection spectra and the maximum pressure amplitude within the redesigned central unit are demonstrated in the upper panel of Figure 1f. A flat reflection curve is observed at ≈3786 Hz corresponds to the absence of a resonant peak in the pressure response. Meanwhile, the quasi‐BIC, denoted by the reflection dip at 3854.6 Hz, exhibits an extremely narrow resonant bandwidth. Unlike general high‐Q resonances, a quasi‐BIC can be traced back to a pure BIC. As shown in the lower half of Figure 1f, the process of transforming a quasi‐BIC into a pure BIC can be systematically traced by introducing dilation to the central solid scatter in the simulation. This dilation is achieved by expanding the solid boundary, characterized by the boundary offset value Δ*t*, which is varied incrementally from 0 to 0.6 mm. As the offset value increases, the resonance dip in the reflection spectrum shifts, eventually leading to the occurrence of pure BIC at 3786 Hz.

**Figure 2:**
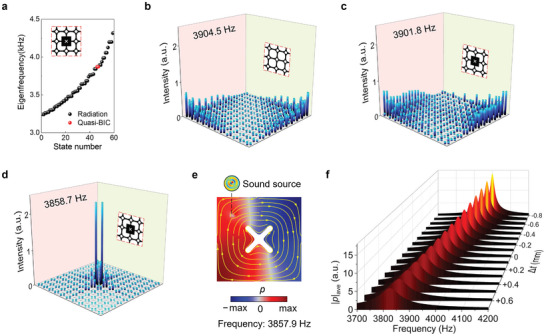
Acoustic field enhancement in open system. a) Eigenmode analysis. b) Sound intensity patterns for the radiation mode at 3904.5 Hz in the superstructure composed of 11 × 11 initial units, c) the radiation mode at 3901.8 Hz, and d) the quasi‐BIC at 3858.7 Hz in the superstructure with the redesigned central unit, respectively. e) Acoustic pressure field (indicated by color scale) and intensity field (indicated by yellow streamlines) of the central unit when a dipole point source is applied (circle dot). f) The average pressure amplitude within the central unit varying the scatter size represented by the boundary offset value Δ*t*.

A box‐shaped superstructure (see Figure 7d in Section 4.2.) is modeled to simulate sound trapping and field enhancement in an open system. **Figure** **2**a presents the eigenmode analysis of the optimized superstructure. The radiation modes ≈3900 Hz for both the initial model and redesigned models are depicted in Figure 2b,c, respectively. Each column represents the average sound intensity within a cavity, with the connecting channels omitted for clarity. Figure 2d highlights a pronounced localization of the acoustic field at 3858.7 Hz, which is indicative of a quasi‐BIC. This strong localization contrasts sharply with the diffuse acoustic fields seen at nearby eigenmodes. To excite this resonant mode, a dipole point source is positioned at the upper left of the central unit. The source was defined by a dipole moment vector (*D_x_*, *D_y_*) = (cos*θ*, sin*θ*) N m^−1^, where *θ* = 0.22*π*. This oblique orientation of the dipole moment introduces asymmetry to the system, breaking its perfect mirror symmetry. The resulting pressure field and intensity field at the resonance frequency of 3857.9 Hz, shown in Figure 2e, demonstrate significant localization with minimal energy leakage, evidenced by the confined pressure distribution and vortex pattern of intensity flux. The resonant frequency can be tuned by introducing erosion (boundary offset Δ*t* < 0) or dilation (Δ*t* > 0) to the boundary of the X‐shaped solid scatter. Figure 2f illustrates the average pressure amplitude within the central unit cell varying the scatter size. The pronounced peak at the resonant frequency underscores the high‐Q nature of the quasi‐BIC. The sensitivity of this resonant mode to variations in scatter size highlights the tunability of the quasi‐BIC, precise control over the resonant frequency and field enhancement.

**Figure 3:**
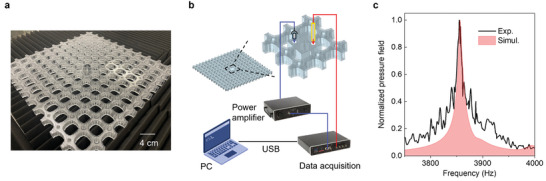
Experimental demonstration of sound pressure enhancement. a) Photograph of the fabricated sample. b) Schematic of the experimental set‐up. The mini speaker and microphone, as the sound generator and detector, are inserted into the cover sheet of the central unit. c) Frequency dependence of normalized pressure field within the central unit. The black solid line (red pattern) represents the experimental (simulation) results.

A 3D‐printed sample, depicted in **Figure** **3**a, has been fabricated to experimentally validate our design's capability to trap and amplify sound through the high‐Q resonance. The experimental setup, illustrated in Figure 3b, involves a micro‐speaker and a microphone within the cover sheet of the central unit, functioning as the sound generator and detector, respectively. Detailed settings can be found in the Section 4.3.

The frequency‐dependent pressure response, shown in Figure 3c, demonstrates a substantial enhancement in localized pressure at the resonant frequency. To facilitate comparison, the measured pressures were normalized to their peak value at 3855 Hz, while the simulation results were normalized to the peak at 3857 Hz. The resonant bandwidths (FWHM) for the pressure enhancement in experimental and simulation results are 17 Hz (from 3845 to 3862 Hz) and 15 Hz (from 3850 to 3865 Hz), resulting in Q‐factors of 226.8 and 257.1, respectively. The measured response curve exhibits a resonant peak at a slightly lower frequency with a broader FWHM compared to the simulation. These discrepancies are likely due to manufacturing imperfections. As demonstrated in Figure 2f, the dilation of the solid scatter can lead to a reduction in resonance frequency and an increase in bandwidth. The fabricated sample may have minor geometric deviations or surface roughness that are not accounted for in the idealized simulation model. Despite these minor differences, the overall agreement between the simulation and experimental results underscores the effectiveness of the proposed topology optimization method for designing BIC structures with high‐Q resonances. At the resonant frequency of 3855 Hz, the pressure measured within the central unit cell of the metastructure is 12.43 times higher than the pressure outside the metastructure. This corresponds to a remarkable pressure amplification of 21.89 dB. This significant increase in localized pressure confirms the presence of the quasi‐BIC and its effectiveness in achieving high‐Q field enhancement.

**Figure 4:**
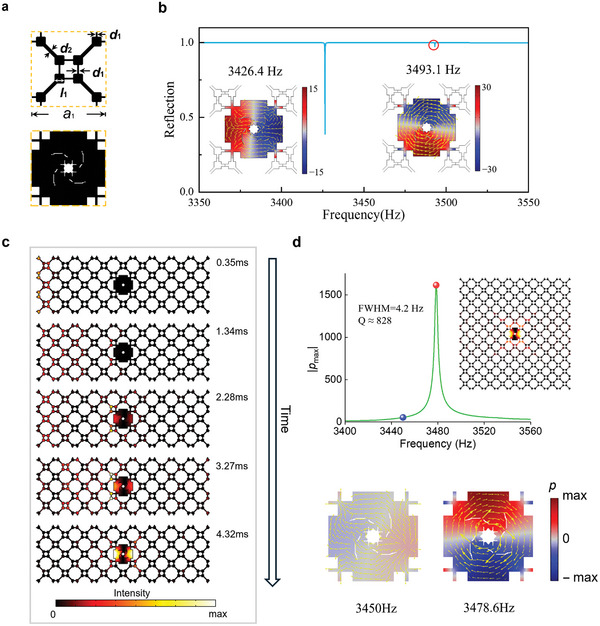
BICs by TO. a) Basic unit cell and the optimized central unit. b) Reflection spectra and resonant field patterns at 3426.4 and 3493.1 Hz, respectively. c) Simulation snapshots of the acoustic intensity distributions at *t* = 0.35, 1.34, 2.28,3.27, and 4.32 ms. d) Upper: frequency response of the maximum pressure magnitude with the central unit when an internal point source is applied. The inset shows the intensity field at the resonant frequency of 3478.6 Hz. Lower: pressure fields (indicated by color scale) and acoustic intensity fields (indicated by yellow arrows) at 3450 and 3478.6 Hz, corresponding to the radiating mode (blue marker) and trapped mode (red marker).

To further demonstrate the versatility of the proposed TO method in achieving high‐Q confinement without human intervention during the design process, we modified the input parameters of the surrounding unit cells. **Figure** **4**a presents a basic unit cell derived from the zone folding of the unit cell in Figure 2a.^[^ ^57^, ^58^ ^]^ In this configuration, the lattice constant is set to *a*_1_ = 56.6 mm, with eight identical square cavities, each having a side length of *l*_1_ = 7.1 mm. The widths of the connecting channels are *d*_1_ = 2.2 mm and *d*_2_ = 2.3 mm, respectively. In this case, no filtering scheme is applied (i.e., the minimum size of the optimized design would be one element, ≈0.44 mm), and a *C*_4_ rotational symmetry constraint is imposed. More details can be found in Section 4.1. The TO‐redesigned central unit cell is depicted in the lower panel of Figure 4a.

A ribbon‐shaped superstructure (see Figure 7c in Section 4.2.) is formed using the redesigned central unit cell surrounded by the basic cells to facilitate the calculation of far‐field reflection. Eigenfrequency analysis confirms two resonant modes with complex frequencies of 3426.4 + 0.07*i* and 3493.1 + 0.0*i*Hz. The reflection spectra, shown in Figure 4b, reveal a narrow bandwidth at 3426.4 Hz corresponding to a quasi‐BIC and a vanished reflection dip at 3493.1 Hz. A time‐dependent simulation has been performed with harmonic sine waves at a frequency of 3426.4 Hz applied through input tubes on the left side of the model. The results are illustrated in Figure 4c. Over time, the acoustic energy gradually concentrated in the central region, with maximum localization observed at *t* = 4.32 ms. This gradual trapping of acoustic energy illustrates the system's capability to localize sound waves effectively, confirming the presence of a quasi‐BIC under far‐field excitation.

To investigate the sound trapping effect, we simulate a box‐shaped superstructure with outgoing boundaries (Figure 7e in Section 4.2.) to study sound trapping and near‐field enhancement when an internal point source was applied. Figure 4d shows the frequency response of the maximum pressure amplitude within the central unit cell. At 3450 Hz, the pressure and intensity fields reveal that acoustic energy radiates outward from the central unit, indicating poor energy trapping. However, at 3478.6 Hz, the fields show perfect energy confinement and amplification within the unit cell. The calculated Q‐factor for this pressure localization is ≈828, demonstrating superior 0D pressure confinement. The ability of the structure to trap and amplify sound energy at a specific resonant frequency highlights its potential for applications in acoustic devices requiring high‐Q resonances, such as sound isolators or energy harvesters.

## 3 Conclusion

In this study, we present a novel design methodology utilizing topology optimization to achieve high‐Q resonances in acoustic metamaterials. By systematically optimizing the central unit cells, our approach substantially enhances field localization and energy trapping, as validated through both simulations and experimental validation. Remarkably, the experiment achieved a maximum pressure amplification of 21.89 dB at a resonant frequency of 3855 Hz, with a Q‐factor of 226.8, closely aligning with simulation predictions. The proposed method's effectiveness is further highlighted by a novel design featuring a chiral central unit cell, derived from a modified initial unit cell, which attained a significantly higher Q‐factor of 828. This capability to confine and amplify acoustic energy with minimal leakage underscores the quasi‐BIC's potential for enhanced wave energy harvesting.^[^ ^59^ ^]^ While the proposed topology optimization approach is effective in numerically designing high‐Q resonances, it does not provide direct insights into the physical nature of the resultant quasi‐BICs. Our findings do not fit neatly into commonly recognized BIC categories (e.g., symmetry‐protected, Friedrich‐Wintgen, or accidental BICs),^[^ ^4^ ^]^ and further investigation is suggested to elucidate their underlying mechanisms.

The TO‐enabled design strategy offers transformative advantages for advanced acoustic applications such as acoustic filtering, sound manipulation, and noise control. By providing a deterministic and systematic framework for exploring the design space, TO generates numerical designs tailored to specific performance requirements. Its inherent flexibility supports the inclusion of diverse constraints, objectives, and performance criteria, making it particularly effective for the design of high‐performance acoustic devices. This is especially valuable in applications such as medical diagnostics, ultrasonic imaging, and non‐destructive testing, where precise control over acoustic properties is critical. Furthermore, the proposed TO framework can be extended to multi‐dimensional wave confinement beyond 0D localization and adapted for various physical domains, such as photonic or plasmonic systems to enable high‐quality light control. This capability can open pathways to a broad range of applications, including sensing and lasing. Additionally, by generating diverse and high‐quality datasets, TO can address one of the key limitations faced by AI methods: the scarcity of reliable training data. The synergy between TO and AI can open the door to new hybrid design methodologies, where TO informs and improves AI, ultimately leading to more advanced, data‐driven tools that can explore design spaces with greater accuracy and efficiency.

## 4 Experimental Section

### Design

The central unit cell embedded within the periodic metamaterial was redesigned using the floating projection topology optimization (FPTO) method, which was a mathematical approach to achieving the best material distribution within the design domain for the specific objective. The acoustic wave propagating problem governed by the Helmholtz equation could be transformed into a typical eigenvalue problem.^[^ ^60^ ^]^ The center unit cell was selected as the design domain. Based on the finite element method, the design domain was discretized with 128 × 128 four‐node square elements, and each element was assigned a design variable, *x_e_* ∈ {0,  1}, where *x_e_* = 0 or 1 represents two different statuses of an element, solid or air, respectively. As those elements could be either 0 or 1 for optimization, the resulting unit cell structure could have any configurations.

The Q‐factor was characterized by *Q_ω_* = Re(*ω_k_*)/2Im(*ω_k_*). Thus, to maximize the Q‐factor, the optimization problem could be formulated as minimizing the imaginary part of the frequency of the target eigenmode while its real part was constrained within a specific range. This optimization problem could be expressed in the following form,

(1) min:fxe=Imωk

(2) s.t.:K−ω2Mp=0ωlow≤Reωk≤ωuppxe=0or1,e=1,2,…

where ***K*** and ***M*** are global stiffness and mass matrices. The TO design approach could be applied with any basic unit cells featuring degeneracy at the high symmetric points of the first Brillouin zone. For example, as indicated by the complex eigenfrequency band diagram in Figure 1b, the initial unit cell had a twofold degeneracy at *f*_1 _= 4300 Hz. In order to achieve a high‐Q resonance ≈3600 Hz, the constraint function was formulated to confine the target mode within the frequency range of 3827 to 3870 Hz. The material properties of each element were interpolated with respect to the design variable as

(3) $$\left\{\begin{array}{c} \frac{1}{B\left(x_{e}\right)}=\frac{1}{B_{a}}x_{e}\\ \frac{1}{\rho \left(x_{e}\right)}=\frac{1}{\rho_{a}}x_{e} \end{array}\right.$$

where *B_a_* = 0.14 MPa and *ρ_a_* = 1.21 kg m^−3^ are the bulk modulus and mass density for air, respectively. It could be seen that *B*(*x_e_*) and ρ(*x_e_*) values became extremely large as *x_e_* tended to a very small value of *x_min_*  =  10^−9^ for solid to prevent singularity. To simulate the radiating boundaries, an imaginary term 0.001*i* was applied to design variables allocated in the outer layer of unit cells.

**Figure 5:**
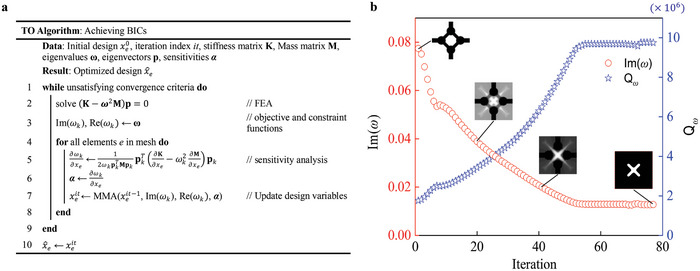
Achieving high‐Q resonances by TO. a) TO algorithm for seeking the optimal configurations supporting BICs. b) Evolution histories of the objective value, the Q‐factor, and topology of the central unit cell.

The topology optimization algorithm was coded with MATLAB as outlined in **Figure** **5**a. In general, the optimization would iteratively update the design variables based on the sensitivity analysis while satisfying the specified constraints. The sensitivity could be computed by the partial derivative of an eigenfrequency with respect to the design variables as

(4) $$\frac{\partial \omega_{k}}{\partial x_{e}}=\frac{1}{2\omega_{k}p_{k}^{T}Mp_{k}}p_{k}^{T}\left(\frac{\partial K}{\partial x_{e}}\;-\;\omega_{k}^{2}\frac{\partial M}{\partial x_{e}}\right)p_{k}$$

where ***p***_k_ is the eigenvector corresponding to the eigenvalue *ω*_k_. Note that the results of Equation (4) are complex numbers, where the imaginary part and real part would be used for calculating sensitivities of the objective and constraint functions, respectively. To achieve a final topology following specific symmetries, such as the rotational symmetries (*C*_2_, *C*_4_) and mirror symmetries along the *x*‐axis and *y*‐axis, the corresponding symmetry constraints scheme could be imposed by averaging the sensitivity numbers and design variables.

A filter scheme could be employed in topology optimization to approximately control the smallest feature of an optimized design,^[^ ^61^ ^]^ which could be expressed as

(5) $${\tilde{x}}_{j}=\frac{\sum_{e}w_{je}x_{e}}{\sum_{e}w_{je}}$$

where ${\tilde{x}}_{j}$ represents the filtered variable, and the subscript *j* here, similar to *e*, indicates the numbering of elements in the filter field. The weight factor *w_je_* is determined by the distance between the centers of elements *e* and *j*.

(6) $$w_{je}=\left\{\begin{array}{c} \frac{r_{\min}\;-\;r_{je}}{\sum_{e}\left(r_{\min}-r_{je}\right)}\;e\in \Omega_{C}\\ 0\;e\notin \Omega_{C} \end{array}\;\right.$$

where *Ω_C_* denotes the filter support domain defined by a circle at the center of element *j* with a specified radius, *r*_min_. The filter scheme indicated that the features smaller than the filter diameter, 2*r*_min_, would be excluded from the final design. Although a larger filter radius could make the final design (e.g., Figure 1c) easily fabricated, it would limit the design freedoms and potentially resulting in a sub‐optimal result. Alternatively, the filter scheme could be abandoned, and thus the smallest feature size would be one element. The resulting design (e.g., Figure 4a) could achieve a higher *Q*‐value due to the increased design freedom, but it might pose greater fabrication challenges.

This TO algorithm seeked a solution based on design variables from the last iteration and sensitivities of the objective and constraint functions, by utilizing the method of moving asymptotes (MMA).^[^ ^62^ ^]^ MMA solved the continuous problem by approximating a convex objective function and linearizing constraint functions while ensuring increasing accuracy of the approximation as the solution approaches an optimum. Meanwhile, the floating projection constraint was imposed to simulate 0/1 constraints of design variables given in Equation (2). Due to space limitations, the details of MMA and FPTO methods could be referred to the references^[^ ^62^ ^]^ and,^[^ ^50^, ^60^ ^]^ respectively. Figure 5b shows the evolutionary histories of the minimal imaginary part of the frequency, *Q*‐value, and structural topology in the first example.

### Simulation

**Figure 6:**
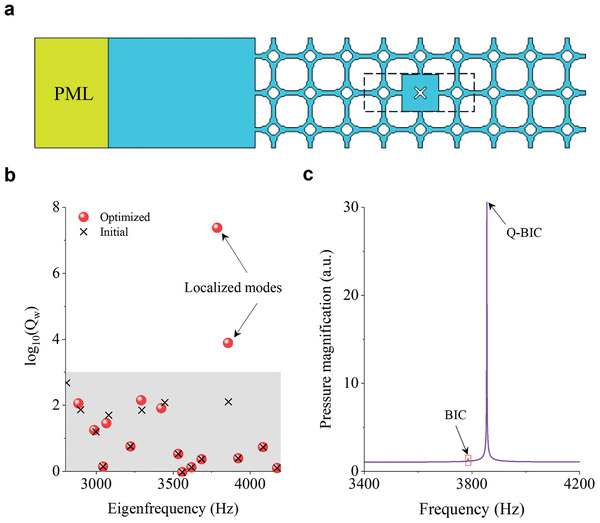
Simulations referring to Figure 1. a) 9 × 3 superstructure. b) Eigenmodes and logarithmic Q‐factors. c) Maximum pressure amplitude within the redesigned unit cell versus incident wave frequencies.

Simulations were performed using finite element analysis with the commercial software COMSOL Multiphysics. **Figure** **6**a illustrates the simulation model, which consisted of a perfectly matched layer and an open domain introduced to the left of a 9 × 3 superstructure, referring to Figure 1d,f. Mirror symmetry was broken along the *y*‐axis while being preserved along the *x*‐axis. Other exterior boundaries on the top, bottom, and right of the model were sound hard boundaries. The simulations were conducted in the Pressure Acoustics module using the thermally conducting and viscous fluid mode. Material properties were defined using the built‐in air model at 20 °C from the COMSOL library.

Eigenfrequency analysis was first performed to confirm the existence of high‐Q resonances introduced by the redesigned unit cell. Figure 6b shows two localized modes with complex eigenfrequencies of 3786.3 + 0.000158*i* and 3855.06 + 0.31*i* Hz, respectively. The corresponding eigen‐field profiles within the dashed box are given in Figure 1d. To observe the frequency response of this metastructure, a background pressure field was applied at the open domain and scanned the frequency from 3400 to 4200 Hz with a step size of 0.5 Hz. The maximum pressure within the redesigned unit cell shown in Figure 6c revealed a sharp peak at 3855 Hz, corresponding to the quasi‐BIC, and a vanishing peak around the BIC frequency of 3786.5 Hz.

**Figure 7:**
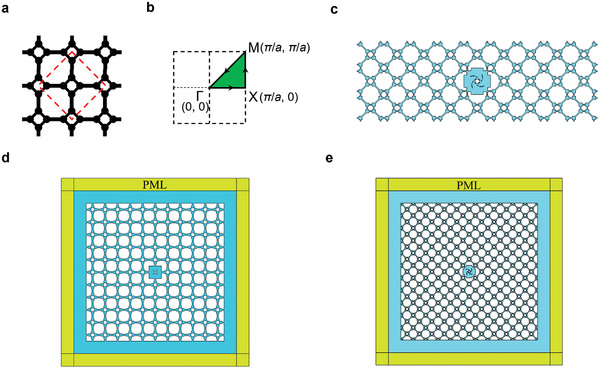
Simulations settings. a) Periodic basic unit cells (3 × 3) referring to Figure 1a, showing the zone folding (red dashed box) for the basic unit cell in Figure 4a. b) Reciprocal space illustrating the first irreducible Brillouin zone. c) Ribbon‐shaped superstructure for response simulations with a far‐field acoustic port. d,e) Box‐shaped superstructure for sound trapping in the central unit cell.

**Figure** **7**a depicts the periodic basic unit cell in the first example (Figure 1a), while the second basic unit cell (Figure 4a) was derived by zone folding, as indicated by the red dashed box, and by transforming circular cavities into square ones. Figure 7b presents the first Brillouin zone for calculating the complex band diagrams of unit cells. Figure 7c illustrates the 9 × 3 ribbon‐shaped superstructure used for reflection and transient analysis, referring to Figure 4b,c. Figure 7d,e displays the simulation models of the 11 × 11 superstructure, corresponding to the results in Figure 2 and Figure 4d, respectively.

### Experiment

The experiment was conducted to validate the effectiveness of this design in trapping sound and enhancing pressure. The sample was made of transparent photosensitive resin via 3D stereolithography printing, as shown in Figure 3a. The fabrication precision was maintained within an error margin of ±0.2%. During the measurement, a mini headphone was mounted on the cover sheet of the central unit cell to provide sound excitations, positioned at the upper left corner as illustrated in Figure 3b. A sine wave sound signal was generated by the built‐in sound card of a BSWA MC3242 data collector and amplified using a BSWA PA50 power amplifier. For data acquisition, an NI 9233 card, paired with an MPA416 microphone installed at the opposite corner to collect the response signal.

## Conflict of Interest

The authors declare no conflict of interest.

## Supporting information



Supporting Information

Supporting Information

## Data Availability

The data that support the findings of this study are available from the corresponding author upon reasonable request.

## References

[1] A. Mujahid , A. Afzal , F. L. Dickert , Sensors. 2019, 19, 4395. 31614484 10.3390/s19204395 PMC6833005

[2] L. Huang , S. Huang , C. Shen , S. Yves , A. S. Pilipchuk , X. Ni , S. Kim , Y. K. Chiang , D. A. Powell , J. Zhu , Y. Cheng , Y. Li , A. F. Sadreev , A. Alù , A. E. Miroshnichenko , Nat. Rev. Phys. 2024, 6, 11.

[3] G. Ma , P. Sheng , Sci. Adv. 2016, 2, e1501595. 26933692 10.1126/sciadv.1501595 PMC4771441

[4] C. W. Hsu , B. Zhen , A. D. Stone , J. D. Joannopoulos , M. Soljačić , Nat. Rev. Mater. 2016, 1, 16048.

[5] J. v. Neuman , E. Wigner , Phys. Z. 1929, 30.

[6] A. A. Lyapina , D. N. Maksimov , A. S. Pilipchuk , A. F. Sadreev , J. Fluid Mech. 2015, 780, 370.

[7] I. Deriy , I. Toftul , M. Petrov , A. Bogdanov , Phys. Rev. Lett. 2022, 128, 084301. 35275659 10.1103/PhysRevLett.128.084301

[8] M. Farhat , Y. Achaoui , J. Martínez , M. Addouche , Y. Wu , A. Khelif , Adv. Sci. 2024, 11, 2402917. 10.1002/advs.202402917 PMC11434233 38962930

[9] S. Liu , S. Huang , Z. Zhou , P. Qian , B. Jia , H. Ding , N. Wang , Y. Li , J. Chen , Phys. Rev. Appl. 2023, 20, 044075.

[10] J. Pan , J. Lu , W. Deng , X. Huang , Z. Liu , Europhys. Lett. 2023, 143, 26002.

[11] Y.‐X. Xiao , G. Ma , Z.‐Q. Zhang , C. T. Chan , Phys. Rev. Lett. 2017, 118, 166803. 28474943 10.1103/PhysRevLett.118.166803

[12] Z.‐D. Zhang , X.‐M. Zhang , S.‐Y. Yu , M.‐H. Lu , Y.‐F. Chen , Phys. Rev. Appl. 2022, 18, 054029.

[13] L. Cao , Y. Zhu , Y. Xu , S.‐W. Fan , Z. Yang , B. Assouar , J. Mech. Phys. Solids. 2021, 154, 104502.

[14] S. An , T. Liu , L. Cao , Z. Gu , H. Fan , Y. Zeng , L. Cheng , J. Zhu , B. Assouar , Phys. Rev. Lett. 2024, 132, 187202. 38759185 10.1103/PhysRevLett.132.187202

[15] S. Vaidya , W. A. Benalcazar , A. Cerjan , M. C. Rechtsman , Phys. Rev. Lett. 2021, 127, 023605. 34296895 10.1103/PhysRevLett.127.023605

[16] H. Qin , Z. Su , M. Liu , Y. Zeng , M.‐C. Tang , M. Li , Y. Shi , W. Huang , C. W. Qiu , Q. Song , Light: Sci. Appl. 2023, 12, 66. 36878927 10.1038/s41377-023-01090-w PMC9988870

[17] D. C. Marinica , A. G. Borisov , S. V. Shabanov , Phys. Rev. Lett. 2008, 100, 183902. 18518374 10.1103/PhysRevLett.100.183902

[18] A. C. Valero , H. K. Shamkhi , A. S. Kupriianov , T. Weiss , A. A. Pavlov , D. Redka , V. Bobrovs , Y. Kivshar , A. S. Shalin , Nat. Commun. 2023, 14, 4689. 37542069 10.1038/s41467-023-40382-y PMC10403603

[19] K. Koshelev , S. Kruk , E. Melik‐Gaykazyan , J.‐H. Choi , A. Bogdanov , H.‐G. Park , Y. Kivshar , Science. 2020, 367, 288. 31949078 10.1126/science.aaz3985

[20] K. Koshelev , S. Lepeshov , M. Liu , A. Bogdanov , Y. Kivshar , Phys. Rev. Lett. 2018, 121, 193903. 30468599 10.1103/PhysRevLett.121.193903

[21] Z. Wang , Y. Liang , J. Qu , M. K. Chen , M. Cui , Z. Cheng , J. Zhang , J. Yao , S. Chen , D. P. Tsai , C. Yu , Photon. Res. 2023, 11, 260.

[22] Y. Liang , D. P. Tsai , Y. Kivshar , Phys. Rev. Lett. 2024, 133, 053801. 39159090 10.1103/PhysRevLett.133.053801

[23] Z. Wang , J. Sun , J. Li , L. Wang , Z. Li , X. Zheng , L. Wen , Adv. Sci. 2023, 10, 2206236. 10.1002/advs.202206236 PMC9982570 36594610

[24] L. Cao , Y. Zhu , S. Wan , Y. Zeng , Y. Li , B. Assouar , Extreme Mech. Lett. 2021, 47, 101364.

[25] S. Mohamed , J. Wang , H. Rekola , J. Heikkinen , B. Asamoah , L. Shi , T. K. Hakala , Laser Photonics Rev. 2022, 16, 2100574.

[26] M.‐S. Hwang , H.‐C. Lee , K.‐H. Kim , K.‐Y. Jeong , S.‐H. Kwon , K. Koshelev , Y. Kivshar , H.‐G. Park , Nat. Commun. 2021, 12, 4135. 34226557 10.1038/s41467-021-24502-0 PMC8257597

[27] Y. Ren , P. Li , Z. Liu , Z. Chen , Y.‐L. Chen , C. Peng , J. Liu , Sci. Adv. 2022, 8, eade8817. 36563161 10.1126/sciadv.ade8817 PMC9788758

[28] B. Duan , S. Liu , X. Liu , X.‐c. Yu , C. Wang , D. Yang , Results Phys. 2023, 47, 106304.

[29] P. Moradi , H. Gharibi , A. M. Fard , A. Mehaney , Phys. Scr. 2021, 96, 125713.

[30] T. C. Tan , Y. K. Srivastava , R. T. Ako , W. Wang , M. Bhaskaran , S. Sriram , I. Al‐Naib , E. Plum , R. Singh , Adv. Mater. 2021, 33, 2100836. 10.1002/adma.202100836 34050568

[31] C.‐L. Zou , J.‐M. Cui , F.‐W. Sun , X. Xiong , X.‐B. Zou , Z.‐F. Han , G.‐C. Guo , Laser Photonics Rev. 2015, 9, 114.

[32] Q.‐Y. Liang , A. V. Venkatramani , S. H. Cantu , T. L. Nicholson , M. J. Gullans , A. V. Gorshkov , J. D. Thompson , C. Chin , M. D. Lukin , V. Vuletić , Science. 2018, 359, 783. 29449489 10.1126/science.aao7293 PMC6467536

[33] L. Xu , K. Zangeneh Kamali , L. Huang , M. Rahmani , A. Smirnov , R. Camacho‐Morales , Y. Ma , G. Zhang , M. Woolley , D. Neshev , A. E. Miroshnichenko , Adv. Sci. 2019, 6, 1802119. 10.1002/advs.201802119 PMC6685498 31406659

[34] Z. Liu , J. Wang , B. Chen , Y. Wei , W. Liu , J. Liu , Nano Lett. 2021, 21, 7405. 34232665 10.1021/acs.nanolett.1c01975

[35] L. Huang , B. Jia , A. S. Pilipchuk , Y. Chiang , S. Huang , J. Li , C. Shen , E. N. Bulgakov , F. Deng , D. A. Powell , S. A. Cummer , Y. Li , A. F. Sadreev , A. E. Miroshnichenko , Phys. Rev. Appl. 2022, 18, 054021.

[36] J. Lee , B. Zhen , S.‐L. Chua , W. Qiu , J. D. Joannopoulos , M. Soljačić , O. Shapira , Phys. Rev. Lett. 2012, 109, 067401. 23006303 10.1103/PhysRevLett.109.067401

[37] L. Huang , B. Jia , Y. K. Chiang , S. Huang , C. Shen , F. Deng , T. Yang , D. A. Powell , Y. Li , A. E. Miroshnichenko , Adv. Sci. 2022, 9, 2200257. 10.1002/advs.202200257 PMC9284153 35561061

[38] L. Huang , Y. K. Chiang , S. Huang , C. Shen , F. Deng , Y. Cheng , B. Jia , Y. Li , D. A. Powell , A. E. Miroshnichenko , Nat. Commun. 2021, 12, 4819. 34376653 10.1038/s41467-021-25130-4 PMC8355331

[39] S. Huang , T. Liu , Z. Zhou , X. Wang , J. Zhu , Y. Li , Phys. Rev. Appl. 2020, 14, 021001.

[40] T. Weber , L. Kühner , L. Sortino , A. B. Mhenni , N. P. Wilson , J. Kühne , J. J. Finley , S. A. Maier , A. Tittl , Nat. Mater. 2023, 22, 970. 37349392 10.1038/s41563-023-01580-7 PMC10390334

[41] Y. Liang , H. Lin , S. Lin , J. Wu , W. Li , F. Meng , Y. Yang , X. Huang , B. Jia , Y. Kivshar , Nano Lett. 2021, 21, 8917. 34459611 10.1021/acs.nanolett.1c02751

[42] J. Li , J. Ren , X. Zhang , J. Opt. Soc. Am. B. 2017, 34, 559.

[43] W. Wang , A. Günzler , B. D. Wilts , U. Steiner , M. Saba , Adv. Photonics. 2023, 5, 056005.

[44] X. Ma , Y. Ma , P. Cunha , Q. Liu , K. Kudtarkar , D. Xu , J. Wang , Y. Chen , Z. J. Wong , M. Liu , M. C. Hipwell , S. Lan , Laser Photonics Rev. 2022, 16, 2100658.

[45] L. Wang , W. Wang , Q. Dong , L. Wang , L. Gao , J. Opt. Soc. Am. B. 2024, 41, A146.

[46] F. Wang , Y. Chen , Z. Zhang , X. Zhang , X. Zhou , Y. Zuo , Z. Chen , C. Peng , Opt. Express. 2023, 31, 12384. 37157399 10.1364/OE.486873

[47] Y. Gao , W. Chen , F. Li , M. Zhuang , Y. Yan , J. Wang , X. Wang , Z. Dong , W. Ma , J. Zhu , Adv. Sci. 2024, 11, 2405750. 10.1002/advs.202405750 PMC11558086 39246128

[48] R. V. Woldseth , N. Aage , J. A. Bærentzen , O. Sigmund , Struct. Multidisc. Optim. 2022, 65, 294.

[49] M. Bendsoe , O. Sigmund , Topology Optimization‐Theory, Methods and Applications, Springer, Berlin 2003.

[50] X. Huang , W. Li , Comp. Meth. Appl. Mech. Eng. 2022, 399, 115444.

[51] H.‐W. Dong , C. Shen , Z. Liu , S.‐D. Zhao , Z. Ren , C.‐X. Liu , X. He , S. A. Cummer , Y.‐S. Wang , D. Fang , L. Cheng , Mater. Today. 2024, 80, 824.

[52] H.‐T. Su , L.‐Y. Wang , C.‐Y. Hsu , Y.‐C. Wu , C.‐Y. Lin , S.‐M. Chang , Y.‐W. Huang , Nano Lett. 2024, 24, 10055. 39047260 10.1021/acs.nanolett.4c01858 PMC11342354

[53] M. Albrechtsen , B. Vosoughi Lahijani , R. E. Christiansen , V. T. H. Nguyen , L. N. Casses , S. E. Hansen , N. Stenger , O. Sigmund , H. Jansen , J. Mørk , S. Stobbe , Nat. Commun. 2022, 13, 6281. 36271087 10.1038/s41467-022-33874-w PMC9587274

[54] Q. Zeng , S. Duan , Z. Zhao , P. Wang , H. Lei , Adv. Sci. 2023, 10, 2204977. 10.1002/advs.202204977 PMC9896075 36504452

[55] W. Li , F. Meng , Y. Chen , Y. f. Li , X. Huang , Adv. Theory Simul. 2019, 2, 1900017.

[56] Z. Xin , L. Zhengyou , M. Jun , L. Youyan , J. Phys.: Condens. Matter. 2003, 15, 8207.

[57] L.‐H. Wu , X. Hu , Phys. Rev. Lett. 2015, 114, 223901. 26196622 10.1103/PhysRevLett.114.223901

[58] Y. Deng , H. Ge , Y. Tian , M. Lu , Y. Jing , Phys. Rev. B. 2017, 96, 184305.

[59] Y. Huang , T. Du , C. Xiang , J. Si , H. Yu , H. Yuan , P. Sun , M. Xu , Int. J. Energy Res. 2022, 2023, 5568046.

[60] W. Li , J. Hu , G. Lu , X. Huang , Eng. Comput. 2024, 40, 2581.

[61] O. Sigmund , J. Petersson , Struct. Multidisc. Optim. 1998, 16, 68.

[62] K. Svanberg , Int. J. Numer. Methods Eng. 1987, 24, 359.

