# Supplementary material for: Topology‐Optimized Bound States in the Continuum with High‐Q Acoustic Field Enhancement
Source: Adv Sci (Weinh). 2025 Mar 27;12(21):2414344. doi: 10.1002/advs.202414344 (PMC12140341; doi:10.1002/advs.202414344)
Supplement: Supplementary file 1 — Supporting Information [file ADVS-12-2414344-s002.docx]

Supporting Information

Topology-optimized bound states in the continuum with high-Q acoustic field enhancement

W. Li, K. Ghabraie, X. Huang*

We have included an animation file (S1_Pressure field and energy flux.gif) in the Supporting Information, illustrating the pressure field and energy flux patterns across excitation frequencies, referring to Figure 2e.
