# Supplementary figures and images for: Topology‐Optimized Bound States in the Continuum with High‐Q Acoustic Field Enhancement
Source: Adv Sci (Weinh). 2025 Mar 27;12(21):2414344. doi: 10.1002/advs.202414344 (PMC12140341; doi:10.1002/advs.202414344)

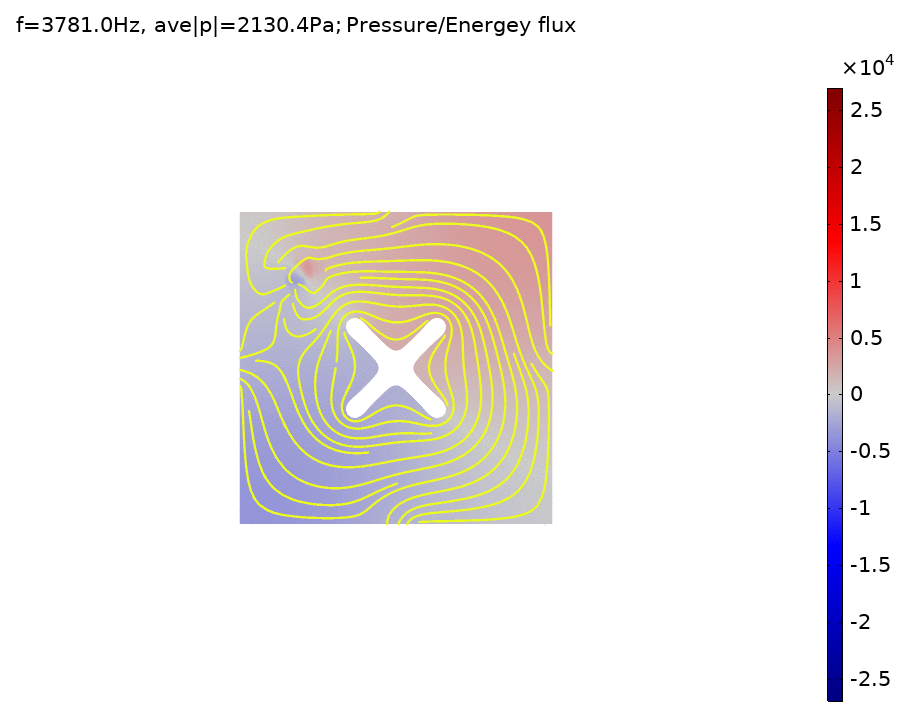

Supplement: Supplementary file 2 — Supporting Information [file ADVS-12-2414344-s001.gif]
